# Intensive Therapeutic Plasma Exchange—New Approach to Treat and Rescue Patients with Severe Form of Yellow Fever

**DOI:** 10.3390/tropicalmed10020039

**Published:** 2025-01-29

**Authors:** Yeh-Li Ho, Youko Nukui, Paula Ribeiro Villaça, Erica Okazaki, Nelson Hidekazu Tatsui, Lucas Chaves Netto, Daniel Joelsons, Tania Rubia Flores da Rocha, Fernanda de Mello Malta, João Renato Rebello Pinho, Aluisio Augusto Cotrim Segurado, Vanderson Rocha

**Affiliations:** 1 Departamento de Infectologia e Medicina Tropical, Hospital das Clinicas, Faculdade de Medicina, Universidade de Sao Paulo, Sao Paulo 05403-000, Braziljoelsons@gmail.com (D.J.);; 2 Serviço de Hematologia, Hemoterapia e Terapia Celular, Hospital das Clinicas, Faculdade de Medicina, Universidade de Sao Paulo, Sao Paulo 05403-000, Brazil; 3 LIM07, Departamento de Gastroenterologia, Hospital das Clinicas, Faculdade de Medicina, Universidade de Sao Paulo, Sao Paulo 05403-000, Brazil; 4 Fundacao Pro-Sangue, Sao Paulo 05403-000, Brazil; 5 Churchill Hospital, Oxford University Hospitals, Oxford OX3 7LE, UK

**Keywords:** yellow fever, flaviviruses, arboviruses, plasma exchange, critical care, acute liver failure

## Abstract

Background: Severe yellow fever (YF) can result in acute liver failure (ALF) and high mortality. The role of therapeutic plasma exchange (TPE) in managing YF-ALF remains unclear. This study evaluated the impact of TPE strategies in severe YF. Methods: This observational case-series study evaluated three groups of patients classified according to treatment: G1 (standard intensive care support [ICS]), G2 (ICS + high-volume-TPE [HV-TPE]), and G3 (ICS + intensive TPE). HV-TPE was performed during 3 consecutive days with extra sessions of one plasma-volume, if necessary, whereas intensive TPE consisted of one plasma volume/session performed twice daily, with additional fresh frozen plasma infusion. Hemostatic agents, including tranexamic acid, platelets, and cryoprecipitate, were administered as needed. TPE was de-escalated based on clinical and laboratory parameters. The primary outcome was mortality. Results: Sixty-six patients were included (G1: 41, G2: 11, G3: 14). Groups had similar baseline characteristics. Mortality was significantly lower in G3 (14%) compared to G2 (82%) and G1 (85%) (*p* < 0.001). Additionally, G3 patients showed a higher frequency of undetectable YF viral load. Conclusions: Intensive TPE is a feasible and effective intervention for severe YF, achieving an 84% reduction in mortality. The limitations of our results are the small sample size, observational and single-center study. Further studies are warranted to elucidate intensive TPE’s role in YF management.

## 1. Introduction

In the last years, yellow fever (YF) outbreaks have occurred in Latin America and Africa. In 2016, an urban YF outbreak in Luanda and Kinshasa resulted in exported cases which reached China via travelers [1]. In 2017, a sylvatic outbreak occurred in the Brazilian Southeast States. From July-2017 to June-2018, 1376 cases were confirmed, with 483 deaths (35%) [2]. During this period, international travelers acquired infections in this area, leading to at least four deaths [3]. The WHO considers urban outbreaks and the spread of disease through international travelers to be real threats [4].

Clinical presentation varies from non-specific mild symptoms to a severe form. Based on current knowledge, the liver is the organ most affected by yellow fever virus (YFV), resulting in acute liver failure (ALF-YF) and fulminant hepatitis [5]. An international normalized ratio (INR, a measure of how rapidly blood coagulates) of >1.5 associated with encephalopathy without preexisting chronic liver disease is the most largely established definition of acute liver failure (ALF) [6].

Intensive care support (ICS) for ALF aims at keeping the patient alive until the liver regenerates enough to restore its normal function. However, in many cases, the intensity of liver injury is so severe that there is no time for its recovery, requiring other therapeutic strategies [7]. Liver transplantation (LT) for ALF has shown success to improve survival [8]. Nevertheless, questions regarding life quality after transplantation, risk of death while waiting for a graft, and consequences of immunosuppression are still points of concern [7]. Additionally, the timing of LT is uncertain. Studies have demonstrated better long-term survival in patients who recovered spontaneously than those who submitted to LT [9].

The Clichy criteria define candidates for LT as patients who present confusion or encephalopathy grades of III-IV and a factor V (FV) of <20% of normal (patients < 30 yo), or a FV of <30% (patients >30 yo) [10]. During the 2017/2018 Brazilian YF outbreak, the Health Ministry adopted “modified Clichy criteria” for LT—i.e., due to quick clinical deterioration, any degree of encephalopathy was considered as a criterion to list patients for LT [11].

Along with LT, other artificial supports are necessary to provide time for liver recovery or a bridge to transplantion in ALF [7,12]. Larsen et al. demonstrated an increased survival rate using a high-volume plasma exchange (HV-TPE) on three consecutive days [13]. The European Association for the Study of the Liver (EASL) recommends therapeutic plasma exchange (TPE) for ALF once it has been shown to improve transplant-free survival and immune modulation [6]. The aim of this study is to describe our experience with TPE in the severe form of YF.

## 2. Material and Methods

### 2.1. Study Population

This is an observational case-series unicentric study evaluating two different TPE strategies for patients with a severe form of yellow fever during the São Paulo, Brazil 2018–2019 yellow fever outbreak. It was conducted at Hospital das Clinicas (HCFMUSP), a 2200-bed tertiary care teaching hospital affiliated to the University of São Paulo with 110 intensive care beds and a 7-bed infectious disease intensive care unit (ID-ICU). On 9 January 2018, the ID-ICU had become the center of expertise for severe forms of YF in SP. Severity criteria for ID-ICU admission were: aspartate aminotransferase (AST) or alanine aminotransferase (ALT) > 3000 U/L; INR > 1.5; platelet count < 90 × 10^9^/L; kidney dysfunction; hemorrhagic manifestations; encephalopathy; or hemodynamic instability [14]. However, in 2019, a yellow fever outbreak occurred in a region of the state of São Paulo where transportation to HCFMUSP takes at least 6 h. For this reason, all patients who had ALT/AST > 500 were referred to HCFMUSP. From 9 Jan to 15 Feb 2018, treatment consisted of standard intensive care support (ICS). In the second half of February 2018, high-volume TPE (HV-TPE) was used in some patients with severe YF in our service. Transient improvement of laboratory results was observed, however hemorrhagic phenomena occurred between sessions of HV-TPE without changing the outcome [15]. Since April 2018, this strategy was modified to more intensive TPE twice a day and supportive transfusion, if necessary. This intensive protocol was used on one patient in 2018 successfully [16], and since then it has been used in the State of São Paulo’s 2019 YF outbreak.

TPE was administered to patients with levels of ammonia > 70 mcmol/L and/or FV < 50%. The definition of the cut-off of ammonia was based on the 2018 experience when patients with ammonia > 70 mcmol/L had a higher relative risk of death (RR 2.0) (data not published). The cut-off of FV < 50% was defined based on Clichy’s criteria, considering a reduction of 30% from the reference value of 70–150% for all patients. In order to compare to previous cases, all patients with ammonia > 70 mcmol/L and/or FV < 50% were selected.

For this analysis, patients were classified into three groups according to type of treatment given: group one (G1)—ICS; group two (G2)—ICS and HV-TPE; and group three (G3)—ICS and intensive TPE. Clinical and laboratory data were extracted retrospectively and stored in the RedCap Web platform.

### 2.2. Therapeutic Plasma Exchange Strategies

HV-TPE strategy (G2): proposed treatment consisted of three consecutive days of plasma exchange. The volume changed was 10% of body weight (bw) with fresh frozen plasma (FFP). One to three additional sessions of one plasma volume each were implemented according to ammonia levels. Supportive transfusion was guided mainly by hemorrhagic episodes.

Intensive-TPE strategy (G3): volume of exchange with FFP was calculated according to bw, hematocrit (Ht), and plasma volume (bw × 0.7 × [1 − Ht]), considering one plasma volume per session. Procedure was performed twice a day, with additional FFP infusion (10 mL/Kg) at night. Platelet concentrate was transfused to keep platelet count > 30 × 10^9^/L or, in the presence of bleeding, >50 × 10^9^/L. In severe or life-threatening bleeds, target was >80–100 × 10^9^/L. Cryoprecipitate transfusion was indicated if fibrinogen level was <100 mg/dL. Based on bleeding as well as FV and/or ammonia levels, the frequency of TPE was tapered to once a day, every other day and discontinuation. During the de-escalation period, FFP was infused (5 mL/Kg) 1–2 times/day, following the same criteria above. Tranexamic acid (10 mg/Kg/dose, IV, every 6–8 h) was prescribed until discontinuation of TPE and topical use was implemented in cases of mucocutaneous bleeding.

TPE was performed using FFP replacement, without anticoagulant (citrate), once all patients had ALF. Calcium chloride was administered at the following rates: for HV-TPE group, 5 mmol per each liter of plasma exchange; for intensive-TPE group, fixed 6.8 mmol of calcium per session. After each session, serum calcium level was assessed, and additional supplementation was considered. COBE Spectra^®^ and Optia^®^ (TerumoBCT, Tokyo, Japan) and COM.TEC^®^ (Fresenius Kabi, Bad Homburg, Germany) TPE devices were employed, using a withdrawal flow of 70 mL/minute on average. In cases where continuous veno-venous hemodialysis was indicated, the plasma exchange system was connected to the return circuit of the dialysis system.

### 2.3. Detection and Quantification of YFV-RNA in Serum Samples

YFV-RNA was isolated from 250 μL of serum samples and 20 μg of RNAse-free glycogen were added to the aqueous phase as an RNA carrier. Real-time quantitative YFV reverse transcription (RT-qPCR) was carried out using SuperScript^®^ III One-Step RT-qPCR System with Platinum^®^ Taq DNA Polymerase (Invitrogen, Thermo Fisher Scientific Brand, Carlsbad, CA, USA). The YFV 5′non-coding region was targeted using specific primers and probe, as previously described [17]. All samples were tested in triplicate.

### 2.4. Assessments and End Points

The primary end point was mortality. Baseline clinical and laboratory aspects were compared among groups.

### 2.5. Statistical Analysis

Survival curves were constructed using the Kaplan–Meier method, and the log-rank test was used to assess differences between curves. Proportional hazard assumption was checked graphically by log-minus-log vs. Log (Time) plot. Comparisons between two groups were assessed using the Student *t*-test, or the Mann–Whitney test if appropriate for quantitative variables, and the Fisher exact test for categorical variables. Assumptions of normality and homogeneity of variance for *t*-test were evaluated.

Differences between the 3 groups in quantitative variables were evaluated by the Kruskal–Wallis test. Comparison of proportions between the 3 groups was performed by using the Fisher-exact test.

Quantitative variables were presented as median (interquartile range [IQR]). Categorical variables were expressed as counts and percentages. Normality was assessed by the Shapiro–Wilk normality test, the D’Agostino–Pearson omnibus normality test, and visual inspection of histograms. All *p*-values were two-sided. Results were considered significant if *p* < 0.05. Statistical analyses were performed using SAS version 9.4 (SAS Institute Inc., Cary, NC, USA) [18].

## 3. Results

### 3.1. Participant Characteristics

During the 2018/2019 yellow fever outbreak in São Paulo, 114 confirmed cases of yellow fever were referred to the ID-ICU of HCFMUSP. Sixty-six patients had levels of ammonia > 70 mcmol/L and/or an FV of <50% and were included for analysis: 41 (62%) were treated with ICS (G1), 11 (17%) received ICS plus HV-TPE (G2) and 14 (21%) received ICS plus intensive TPE (G3).

There were no differences among the three groups related to sex and clinical presentation at admission. All groups had values of AST/ALT > 3000 U/L, however patients of G3 had lower levels of AST/ALT compared to the other groups, probably as a consequence of the change in the transfer criteria, which allowed earlier assistance, preventing the progression of the disease. No differences were observed among the groups with respect to the other laboratory tests (Table 1).

Concerning G3, the patients were admitted with a median of six days of symptoms and fever was noted in 86% of them. Surprisingly, jaundice was present in only 43% of the patients at admission. Hemorrhagic phenomena occurred in 11 patients (79%), the vast majority being gastrointestinal bleeding. Twelve patients (86%) had seizures and/or headaches as neurologic manifestations. One of the female patients (LO) was pregnant and was diagnosed with reactive hemophagocytic syndrome, which was successfully treated with human intravenous immunoglobulin. Further details on the G3 patients are shown in Table 2.

### 3.2. Therapeutic Plasma Exchange

The median volume treated and time per procedure were higher in G2 (*p* = 0.005 and *p* < 0.001, respectively) although G3 had a higher median number of procedures per patient (*p* < 0.001) (Table 1). However, 45% of the G2 patients died before concluding the proposed treatment (three consecutive days).

Transfusion reactions such as transfusion-related acute lung injury, transfusion-associated circulatory overload or ABO incompatibility were not observed. Only mild allergic symptoms occurred during or soon after the procedure. Hypocalcemia (ionic calcium < 4.0 mg/dL) was more frequent in G2 (*p* < 0.001) (Table 1).

### 3.3. Outcomes

**Figure 1:**
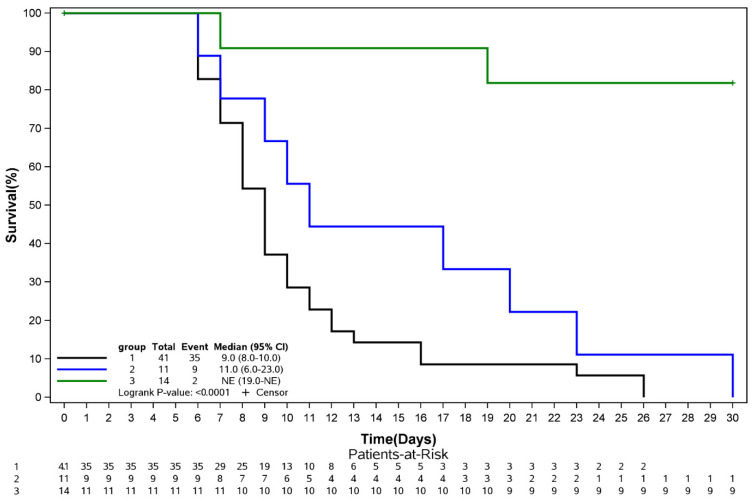
Kaplan–Meier survival estimates during the first 30 days of follow-up according to treatment. Group 1 = intensive care support (ICS); Group 2 = ICS plus high-volume therapeutic plasma exchange (TPE) (HV-TPE); Group 3 = ICS plus intensive TPE.

The mortality rates in G1 and G2 were similar, at 85% and 82%, respectively (Table 1). The mortality rate for patients in G3 was significantly inferior, as demonstrated in the Kaplan–Meier survival curve (Figure 1). Only two patients died in G3 (Table 2).

One was a 48-yo male patient (VPP) who reported long-term use of alcohol, with four days of symptoms. AST and ALT levels were markedly elevated (403 and 148 times greater than the upper limit of normal [ULN], respectively), with high ammonia levels (3.7 times the ULN) and low levels of FV (reduction of 90% from normal). Four different bacteria were identified 48 hs later in admission blood culture (*E. coli*, *S. viridians*, *S. pneumoniae* and *F. nucleatum*). Despite ICS, antibiotics, and intensive TPE, the patient presented with a massive alveolar hemorrhage and died 72 hs after admission (Table 2)

The second was also a 48-yo male patient (VSS), previously vaccinated against YFV (10 years before), with six days of symptoms. AST and ALT levels were markedly elevated (163 and 103 times the ULN, respectively), high ammonia levels (2.3 times the ULN), and normal levels of FV. After five days of intensive TPE, all laboratory results were normalized. However, on the sixth day of admission, *S. aureus* was identified in a blood culture and the patient died seven days later.

**Figure 2:**
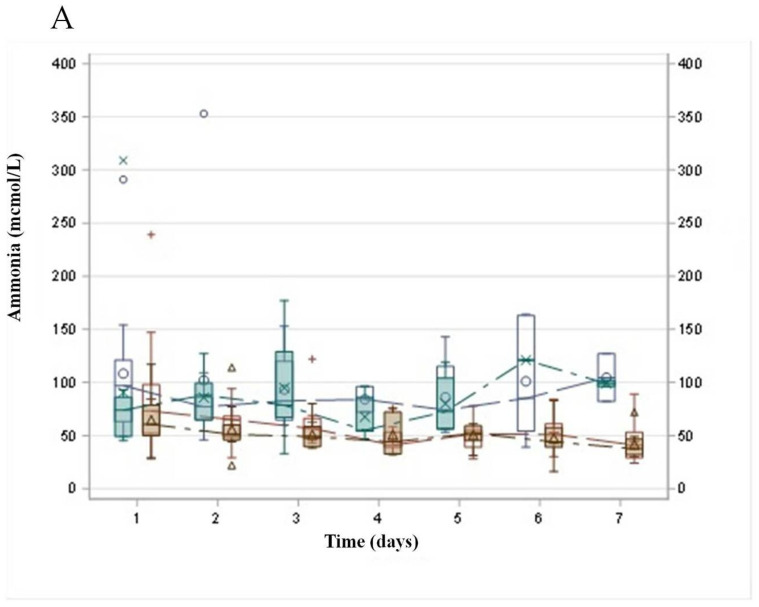

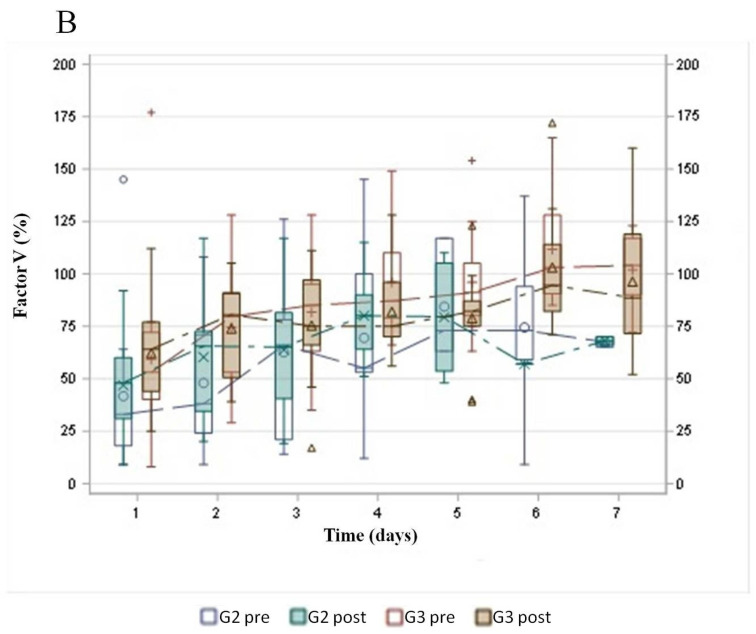
Evolution of median levels of ammonia (**A**) and factor V (**B**) during the first seven days according to therapeutic plasma exchange (TPE). Group 2 (G2) = intensive care support (ICS) plus high-volume therapeutic plasma exchange TPE (HV-TPE) and group 3 (G3) = ICS plus intensive TPE. x: median G2 pre, ◦: median G2 post, +: median G3 pre, ∆: median G3 post.

**Figure 3:**
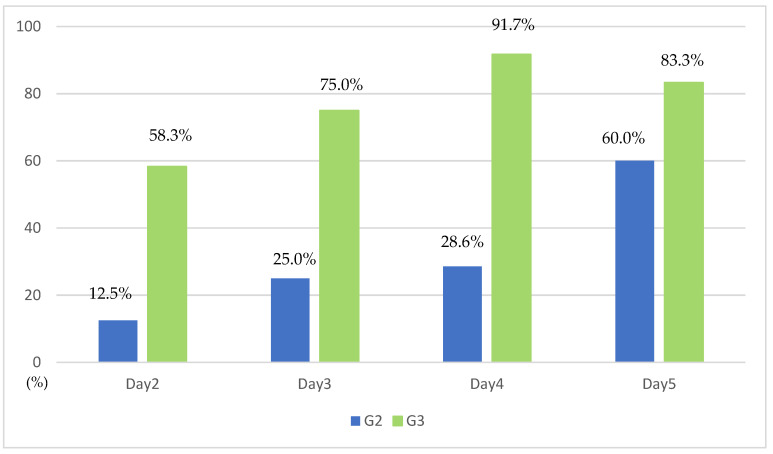
Proportion of undetectable viral load (under 17 copies/mL) during the first five days according to therapeutic plasma exchange (TPE). Group 2 (G2) = ICS plus high-volume therapeutic plasma exchange TPE (HV-TPE); Group 3 (G3) = ICS plus intensive TPE.

Comparing G2 and G3, the evolution of the median levels of ammonia exhibited opposite directions: an increasing tendency for G2 and a decreasing tendency for G3 (*p* = 0.0002). On the other hand, the levels of FV showed a similar increasing tendency in G2 and G3, however, with statistical significancy (*p* = 0.014). (Figure 2A,B). Three days after TPE (day 4), the serum YF viral load became undetectable in 91.7% of G3 compared to 28.6% of G2 (Figure 3).

## 4. Discussion

Recent efforts have been dedicated to developing antiviral therapies against the YFV, but they are still limited to pre-clinical studies [19]. Hence, the reinforcement of surveillance programs and prevention through vaccination are mandatory, including for travelers. It is estimated that millions of unvaccinated travelers visit YF-endemic regions, which creates a risk of illness and spread of the YFV to non-endemic areas [20].

Before the recent Brazilian YF outbreak, most previous epidemics occurred in times in which critical care support was not available or in remote areas with scarce medical assistance. In general, the clinical and laboratory prognostic factors are based on a surveillance database and include all notified cases.

A retrospective cohort study, using the Brazilian National Surveillance System, reported that elevated AST (>1200 U/L) and jaundice were independent risk factors associated with mortality in a multivariate analysis [21]. Due to the high mortality rate during the early YF outbreak in São Paulo, in January-2018 criteria for ICU admission were established, which included higher levels of transaminases among other conditions. In contrast, during the 2019 outbreak, the virus moved to the south of the State, an area with a smaller population and poorer healthcare assistance, leading to the earlier-referral decision. The YFV vaccination campaign was also widely implemented during 2018. As a result, there was a reduction of YF cases, leading to an earlier referral decision.

Recent studies have advanced the understanding of severe yellow fever pathogenesis. Meulen et al. observed that fatal cases of yellow fever exhibited statistically significantly higher levels of IL-6, TNF-α, and IL-1 receptor antagonist (IL-1RA) compared to non-fatal hemorrhagic and severe cases [22]. Furthermore, Gonçalves et al. identified higher levels of B-cell activation markers in fatal cases, suggesting a hyperactive and potentially dysregulated B-lymphocyte response; increased expression of genes related to neutrophil activation in fatal cases; and reduced expression of HLA-II in dendritic cells in fatal cases of yellow fever [23]. These findings suggest immune dysregulation as a key mechanism in severe disease. Additionally, viral load and viral kinetics have also been shown to be independent risk factors for mortality [24,25].

Although TPE is recommended for the management of ALF by the American Society for Apheresis and EASL [6,26], few centers apply this strategy. The rationale is to remove endotoxins, ammonia, bilirubin, and inflammatory mediators among others, thereby allowing hepatic regeneration and recovery [26,27,28]. In addition, through the replacement of plasma factors, TPE improves hemorrhagic episodes. Iwai et al., in a study involving 16 patients with ALF, showed a reduction of TNF-alpha, IL-6, and endotoxins plasma levels after TPE [29].

In a trial enrolling 182 patients, Larsen et al. compared standard medical treatment (SMT) to SMT plus HVP. They showed a reduction of proinflammatory mediators following HVP, leading to the attenuation of innate immune activation and an improvement of multi-organ dysfunction. They could demonstrate, for the first time, a statistically significant benefit of HVP in patients with ALF by increasing liver-transplant-free survival [13]. However, in those studies patients with ALF secondary to the YFV were not included.

The high mortality rates of severe YF forms during the YF outbreak in SP despite intensive care support prompted us to use HV-TPE for YF-ALF [14]. In our casuistic, using a similar large-volume exchange strategy, we could not observe any benefit in mortality. G1 and G2 had similar mortality rates despite the use of TPE. We impute this finding to the fulminant onset and aggressive course of ALF-YF, faster than that observed in other etiologies. This hypothesis was corroborated by the short survival of G1 patients and by the transient improvement of laboratory results observed after HV-TPE in G2.

The close relationship between elevated ammonia levels and the development of encephalopathy is well established [30]. During the first month of the outbreak, we observed that a presence of ammonia > 70 mcmol/L and/or FV < 50% were associated with higher mortality. Among 59 patients with this laboratory profile, the first 48 were treated with ICS or LT, and thirty-nine (81%) of them died. Clemmesen et al., using HV-TPE in 11 patients with ALF and hepatic encephalopathy grade III-IV, demonstrated a reduction of arterial ammonia [31]. Nevertheless, in our casuistic using a similar strategy, we observed an increasing trend of ammonia levels instead. In Larsen’s study, an initial significant decrease of ammonia levels was observed although it was not sustained after day three.

In order to overcome the aggressive behavior of ALF-YF and aiming to obtain safe levels of ammonia, a lower volume of TPE per session and a reduction of the interval between the procedures were implemented (intensive TPE). This approach allowed a sustained decrease of ammonia (Figure 2A, G3), resulting in a higher survival rate.

The studies on HV-TPE in hepatotropic viral diseases are limited. Larsen et al. included only 11 patients with acute viral hepatitis, and six of them submitted to HV-TPE [13]. Only two among 11 patients treated by Clemmesen et al. had viral hepatitis [31]. Likewise, Akdogan et al., using a standard plasma exchange protocol, had only 20% (8/39) cases of viral hepatitis in their casuistic [32]. Unlike drug-induced or toxic hepatitis, in viral etiologies the causal agent is still present and the liver injury persists.

Despite the fact that common knowledge presumes a viremia of five days in YF, recent studies demonstrated longer persistence of the virus in the serum and other organs [33,34]. Additionally, in a cohort of the 2018 SP outbreak, a higher viral load was demonstrated to be an independent predictor factor associated with death [24]. and, as we have shown, intensive TPE was able to remove the virus, thus reducing liver damage, another possible reason to justify the success of intensive TPE in severe yellow fever.

Although transaminase levels in G3 patients were lower than in G2, they remained more than 100 times above the upper limit of normal, underscoring the severity of the disease. Additionally, studies have increasingly demonstrated that the yellow fever virus exhibits tropism for organs beyond the liver, with RNA or antigens detectable in the myocardium, lungs, kidneys, pancreas, and spleen [35,36]. Furthermore, coagulopathy has been shown to occur independently of hepatocellular tropism [37,38]. These findings highlight that the involvement of multiple organs significantly contributes to the severity of the disease, and therefore show the role of TPE in restoring metabolic and immunological balance and replenishing coagulation factors.

Intensive TPE apparently was safer with a lower incidence of hypocalcemia compared to HV-TPE. It was also associated with a reduced median volume treated and shorter time per session, allowing sequential treatment for a major number of patients. The possibility of simultaneous treatment for a large number of patients is particularly important considering the dramatic increase of YF in regions with health resource constraints, and the seasonal pattern of the disease in outbreaks. LT is a recognized approach for the management of ALF, however there is very restricted experience in its use as a treatment for ALF-YF. Moreover, the high cost of LT and the shortage of liver donors curtail the feasibility of the procedure.

Some limitations of our study include the small sample size and the need for randomized trials to confirm the efficacy of this strategy. However, given the fulminant progression and high lethality of the disease, an adaptive pragmatic trial could represent a feasible approach to demonstrate this treatment’s effectiveness.

## 5. Conclusions

We demonstrated that in patients with ALF secondary to YF, intensive TPE associated with guided transfusion is a feasible and effective artificial support to keep patients alive until hepatic regeneration and recovery occurs. This was demonstrated by a reduction of 84% in mortality. Therefore, it is important to share our successful experience, although limited to a small number of cases. Undoubtedly there are many knowledge gaps related to the effects of therapeutic plasma exchange in the course of YF and our limitations of a small sample size, and the observational and single-center nature of this study. Further studies are required to better understand the role of TPE in severe YF.

## Figures and Tables

**Table 1:** Comparison of patients treated with intensive care support versus different regimens of therapeutic plasma exchange.

Characteristics	Group 1 (N = 41)	Group 2 (N = 11)	Group 3 (N = 14)	*p*
Sex: male—n (%)	34 (83)	11 (100)	11 (79)	0.30
Age (yr)—median (IQR)	51 (30–58)	37 (22–60)	39 (25–48)	0.021
**Clinical presentation at admission**				
Interval symptoms—admission (days); (IQR) ^a^	5 (5–7)	6 (3–7)	6 (4–7)	0.87
Hemorrhagic manifestation ^b^—n (%)	29 (71)	8 (73)	11 (79)	0.93
Seizures—n (%)	12 (29)	2 (18)	5 (36)	0.63
**Laboratory test at admission (reference values); median (IQR)**				
AST (female < 31 U/L; male < 37 U/L)	7001 (7000–12,968)	12,727 (8602–18,151)	6016 (5001–8682)	0.0075
ALT (female < 31 U/L; male < 41 U/L)	5129 (3465–7000)	6085 (3947–9544)	3059 (2041–4479)	0.019
Ammonia (11–32 mcmol/L)	98 (66–155)	97 (79–106)	74 (57–98)	0.28
Factor V (70–150%)	30 (16–43)	26 (16–42)	45.5 (25–69)	0.28
Total bilirubin (0.20–1.00 mg/dL)	5.88 (4.49–7.77)	4.88 (1.52–6.69)	5.07 (4.32–6.26)	0.33
Fibrinogen (150–400 mg/dL)	91 (72–125)	120 (89–165)	115 (69–130)	0.18
PT—INR (>1.5)—n (%)	37 (90)	10 (91)	11 (79)	0.49
Platelet count (140–450 × 10^9^/L)	76 (47–91)	78 (53–104)	55.5 (44–79)	0.28
**Characteristics of TPE**				*p* (G2xG3)
Volume treated (mL/Kg) per procedure per patient—mean + SD	NA	60.26 + 16.58	43.83 + 5.58	0.005
Time per procedure (minutes)—mean + SD	NA	143.1 + 46.23	66.62 + 11.18	<0.001
Number of procedures per patient—median (IQR)	NA	5 (2.0–5.0)	11.5 (10–15.0)	<0.001
Adverse event: n (%)				
Patients with allergic reaction	NA	1 (9.1)	1 (7.1)	>0.99
Number of procedures with hypocalcemia (ionic calcium < 4.0 mg/dL)	NA	17/41 (41.5%)	18/175 (10.3%)	<0.001
Outcome				
Death—n (%)	35 (85)	9 (82)	2 (14)	<0.001

Abbreviations: AST, aspartate aminotransferase; ALT, alanine aminotransferase; PT, prothrombin time–INR, international normalized ratio. NA = not applicable. Group 1 (G1) = intensive care support (ICS); Group 2 (G2) = ICS plus high-volume therapeutic plasma exchange TPE (HV-TPE); Group 3 (G3) = ICS plus intensive TPE. ^a^ N = 40, group 1. ^b^ Hemorrhagic manifestation includes melena, hematemesis, gum bleeding, petechiae, hemoptysis, and oozing from sites of catheters.

**Table 2:** Characteristics of patients of group 3 (G3—Intensive care support associated with intensive TPE) at admission and outcome.

Characteristics/Patient	EAC	RBQ	ID	VPP	JTSLC	MV	VCM	ERD	JAM	JCC	LO ^a^	VSS	BMA	TMGS	Median (Min–Max)/Frequency (%)
Sex	Male	Female	Male	Male	Male	Male	Male	Male	Male	Male	Female	Male	Male	Female	Male—11 (79%)
Age (yr)	43	27	48	48	28	59	25	18	55	49	34	48	22	17	39 (17–59)
**Clinical presentation**															
Interval symptoms—admission (d)	7	6	4	4	3	4	6	2	7	8	13	6	6	7	6 (2–13)
Fever	Yes	Yes	Yes	Yes	Yes	Yes	Yes	Yes	No	Yes	Yes	No	Yes	Yes	12 (86%)
Jaundice	No	Yes	Yes	Yes	No	No	No	No	Yes	No	No	Yes	No	Yes	6 (43%)
Nausea and/or vomiting	Yes	Yes	Yes	Yes	Yes	Yes	Yes	Yes	Yes	Yes	Yes	Yes	Yes	Yes	14 (100%)
Abdominal pain	Yes	Yes	No	Yes	Yes	Yes	No	No	Yes	Yes	No	Yes	Yes	Yes	10 (71%)
Hemorrhagic manifestation	Melena, hematemesis	Gum bleeding, melena	Gum bleeding	Hematemesis	No	Melena	No	Hematemesis petechiae	Gum bleeding	No	Gum bleeding	Melena	Hematemesis hematuria	Muscle hematoma left arm	11 (79%)
Neurologic manifestation	Headache	Seizure	Headache seizure	Headache	Headache	Headache	No	No	Seizure	Headache	Headache	Headache	Headache, delirium, somnolence, seizure	Seizure	12 (86%)
**Laboratory test (normal range)**															
AST (female <31 U/L; male <37 U/L)	8477	6495	4063	14,913	5503	3815	5317	9955	6000	3133	5001	6033	9912	8682	6017 (3133–14,913)
ALT (female <31 U/L; male <41 U/L)	4479	1954	2041	6062	2650	1938	3375	4755	3759	2740	1566	4242	7597	2612	3059 (1566–7597)
Ammonia (11–32 mcmol/L)	98	71	74	117	51	56	84	77	239	57	74	72	55	119	74 (51–239)
Factor V (70–139%)	69	50	72	11	25	8	46	35	44	45	73	69	8	50	45.5 (8–73)
Fibrinogen (150–400 mg/dL)	45	58	130	130	265	129	120	190	68	85	69	107	116	114	115 (45–265)
PT—INR (0.95–1.20)	1.80	2.10	1.22	3.10	1.35	1.31	1.78	1.80	1.67	2.00	1.96	1.62	2.89	1.6	1.79 (1.22–3.10)
Platelet count (140–450 × 10^9^/L)	56	61	22	94	112	36	73	133	55	79	51	39	44	55	55.5 (22–133)
**Outcome**	Alive	Alive	Alive	Death	Alive	Alive	Alive	Alive	Alive	Alive	Alive	Death	Alive	Alive	Alive—12 (86%)

^a^ 31st week of gestation + reactive hemophagocytic syndrome.

## Data Availability

All data presented in this study are available upon request to the corresponding author.
